# Should treatment decisions in septic arthritis of the native hip joint be based on the route of infection?

**DOI:** 10.5194/jbji-8-209-2023

**Published:** 2023-10-12

**Authors:** Fred Ruythooren, Stijn Ghijselings, Jordi Cools, Melissa Depypere, Paul De Munter, Willem-Jan Metsemakers, Georges Vles

**Affiliations:** 1 Department of Orthopaedic Surgery, University Hospitals Leuven – Gasthuisberg, Leuven, Belgium; 2 Institute for Orthopaedic Research and Training (IORT), Catholic University Leuven, Leuven, Belgium; 3 Department of Laboratory Medicine, University Hospitals Leuven – Gasthuisberg, Leuven, Belgium; 4 Department of General Internal Medicine, University Hospitals Leuven – Gasthuisberg, Leuven, Belgium; 5 Department of Microbiology, Immunology and Transplantation, Laboratory for Clinical Infectious and Inflammatory Disorders, KU Leuven, Leuven, Belgium; 6 Department of Traumatology, University Hospitals Leuven – Gasthuisberg, Belgium; 7 Department of Development and Regeneration, KU Leuven, Leuven, Belgium

## Abstract

**Background**: Surgical management of septic arthritis (SA) of the hip aims at treating the infection by either preserving, resecting or replacing the joint. In some cases, joint preservation should be attempted, whereas other cases would benefit from immediate joint resection or replacement. Prognostic factors have been proposed to guide decision-making. We hypothesized that most of these factors can be simplified to three subgroups based on the route of infection: contiguous spreading, direct inoculation or hematogenous seeding. **Methods**: A total of 41 patients have been treated surgically for SA of the native hip at our tertiary hospital during the last 16 years. Medical records were studied, and various patient and disease characteristics were collated. **Results**: Significant differences between (1) level of fitness, (2) condition of the hip joint, (3) micro-organisms and (4) chance of femoral head preservation were found for patients with SA of the native hip resulting from the three aforementioned subgroups. Femoral head resection was necessary at one point in 85 % of patients. Patients with hematogenous infections of undamaged hips had a reasonable chance (53 %) of avoiding joint resection or replacement. Hip arthroplasty was performed on 46.3 % of patients, with an infection rate of 10.5 %. **Conclusion**: Patients with SA of the native hip resulting from contiguous spreading, hematogenous seeding or direct inoculation differ significantly and should be considered distinct clinical entities. Route of infection is directly related to the chance of femoral head preservation and should, therefore, guide decision-making. Only patients with hematogenous infection to a previously healthy hip had the possibility of femoral head preservation.

red.ruythooren@student.kuleuven.be]FredRuythooren eorges.vles@uzleuven.be]GeorgesVles

## 1 Introduction

Septic arthritis (SA) of the native hip is a rare orthopaedic emergency that can have life-altering consequences (Kao et al., 2019; Mathews et al., 2010). Treatment can be challenging due to delayed diagnosis, pre-existing joint disease and the specific anatomical construction of the hip joint, the latter of which requires surgical techniques that preserve blood supply to the femoral head (Lum et al., 2018). The lack of high-powered studies and well-accepted guidelines further hampers clinical decision-making. Surgical treatment modalities aim at preserving, resecting or replacing the native hip. In recent years, both arthroscopic debridement and lavage as well as immediate one- or two-stage total hip arthroplasty (THA) have gained popularity (Anagnostakos et al., 2016; Balato et al., 2021; Blitzer, 1993; D'Angelo et al., 2021; Fukushima et al., 2021; Huang et al., 2010; Lee et al., 2014; Lum et al., 2018; Manadan and Block, 2004; Papanna et al., 2017; Ravn et al., 2023; Mathews et al., 2007). Although acceptable results have been reported for these vastly different approaches, it is likely that joint preservation should be attempted in some patients, whereas other patients would benefit from immediate joint resection or (staged) replacement (Hipfl et al., 2023; Tan et al., 2021). Femoral head resection may be indicated in cases with severe pre-existing damage to the joint or in cases where one believes that control over infection cannot be achieved because of micro-organisms residing in avascular cartilage (Lum et al., 2018). Therefore, several studies have tried to identify factors associated with the need for repeat surgery and the inability to preserve the native hip joint. This has resulted in a wide range of sometimes contradicting factors related to the host, the joint and the micro-organism(s) involved, as displayed in Table 1 (Bauer et al., 2010; Huang et al., 2020; Hunter et al., 2015; Kao et al., 2019; Khazi et al., 2020; Mabille et al., 2021; Matthews et al., 2008; Tan et al., 2021; Kim et al., 2023). We hypothesized that most of these risk factors are associated with and can be simplified to the three different routes of infection, i.e. SA of the native hip resulting from contiguous spreading (CS), hematogenous seeding (HS) or direct inoculation (DI) (Shirtliff and Mader, 2002).

**Table 1:** Identified risk factors associated with certain outcomes within the treatment of SA of native hip joints.

Publication	Risk factor	Outcome
Matthews et al. (2008)	Symptom duration of over 3 weeks prior to hospital presentation	Necessity of excision arthroplasty
Bauer et al. (2010)	No clinical, microbiological or treatment-related criteria emerged as risk factors	Reinfection after one- or two-stage TJA
Hunter et al. (2015)	Diabetes, inflammatory arthropathy, involvement of a large joint, synovial cell count exceeding 85 000 cells per litre and *S. aureus* cultured	Failure of a single surgical debridement
Kao et al. (2019)	Concurrent infections and liver cirrhosis	Poor outcome (e.g. mortality or disease recurrence)
Tan et al. (2021)	Presence of antibiotic-resistant organisms, male gender, diabetes and post-surgical cause (e.g. ORIF)	Development of PJI after TJA in patients with prior native joint SA.
Khazi et al. (2020)	Preoperative septicemia or septic shock	Return to operating room within 30 d
Huang et al. (2020)	Age, male sex and comorbidities	Mortality
Mabille et al. (2021)	Age and male sex	Failure of initial treatment
Kim et al. (2023)	Systemic inflammatory disease, CCI, preoperative albumin, preoperative haemoglobin, time between symptom onset and admission >7 d, and socioeconomic deprivation	Lower complications and repeatwashout; 1-year mortality and repeat washout

The abbreviations/acronyms used in the table are as follows: ORIF – open reduction and internal fixation; PJI – prosthetic joint infection; TJA – total joint arthroplasty; CCI – Charlson comorbidity index.

Thus, our primary aim was to determine if SA of the native hip resulting from contiguous spreading, hematogenous seeding or direct inoculation should be considered three distinct clinical entities. Secondary aims were to identify other prognostic factors, to see if there is a subgroup of patients in whom preservation of the femoral head should be attempted and to assess outcomes of THA in this subgroup of patients.

## 2 Methodology

### 2.1 Design

This retrospective study was approved by the medical ethics committee of University Hospitals Leuven (reference no. S64930). Medical records of all patients aged 18 years and older for whom synovial fluid and/or tissue samples were obtained between 1 January 2005 and 1 January 2021 were screened (resulting in 13 764 samples screened). Patients were eligible for inclusion if they met the criteria for SA below. The presence of an artificial joint resulted in immediate exclusion. Moreover, patients who did not receive adequate treatment because they refused or for whom the risk of surgery was considered too high were excluded. All included patients received surgical intervention combined with targeted antibiotic regimes. At least one surgical intervention had to be performed at the University Hospitals Leuven (Belgium); thus, in some cases, initial surgical debridement was performed at a referring hospital.

### 2.2 Definitions

Diagnosis of SA of the native hip was made if (1) the aerobic or anaerobic cultures from tissue or fluid samples obtained from hip arthrocentesis or arthrotomy grew microorganisms and/or (2) the synovial cell count exceeded 50 000 white blood cells per microlitre, of which >90 % were polymorphonuclear neutrophils (PMNs) (Margaretten et al., 2007; Ravn et al., 2023; Ross, 2017). Infections resulting from the spread of the pathogen through surrounding soft tissue into the hip joint were considered the result of CS (e.g. decubital wounds or fistula tracts in gastrointestinal pathology). DI infections were caused by the iatrogenic introduction of the pathogen directly into the hip joint (e.g. intra-articular injections or hip-related surgery). HI infections were the result of the pathogen being seeded into the hip joint from a distant source (e.g. endocarditis or bacteremia after primary skin or urinary tract infections).

### 2.3 Variables collected

Data were collected on patient characteristics, past medical history, clinical presentation, laboratory findings, route of infection, imaging findings, microbiology findings, state of the hip joint, and the type and number of surgical procedures performed and their outcomes (Ruythooren, 2023). A full list of collected variables can be found in Table 2.

**Table 2:** Proportion comparison between patient and disease characteristic for all routes of infection.

	Overall	DI	HS	CS	p value
N	41	8	17	16	
Demographics					
Sex (male : female)	31:10	4:4	11:6	16:0	0.0052 ${}^{d}$
Age	57.1 ± 17.4	53.0 ± 14.9	58.1 ± 21.7	58.1 ± 13.8	0.57
BMI >30	5	0	4	1	0.16
Health scores					
CCI	3.2 ± 2.4	2.0 ± 2.3	2.8 ± 2.7	4.2 ± 1.7	0.029 ${}^{d}$
ASA	2.6 ± 0.7	2.0 ± 0.8	2.6 ± 0.6	2.9 ± 0.3	0.0080 ${}^{d}$
Past medical history					
Smoking	10	2	3	5	0.68
Diabetes	5	1	2	2	0.99
HIV	1	0	1	0	0.99
Cancer	9	1	4	4	0.89
Local RTx	4	1	1	2	0.82
SCI	10	0	0	10	0.00010 ${}^{d}$
Immunosuppressants	3	0	3	0	0.21
Inflammatory arthropathy	3	0	3	0	0.21
Presentation					
Decubitus	12	0	0	12	0.00010 ${}^{d}$
Endocarditis	2	1	1	0	0.67
Multi-joint involvement	7	0	6	1	0.034 ${}^{d}$
CRP (mg L ${}^{-1}$ )	115 ± 101.1	66 ± 83.0	132 ± 89.5	121 ± 117.6	0.15
WBC (10 ${}^{9}$ L ${}^{-1}$ )	21 ± 11.9	27 ± 13.1	17 ± 11.3	22 ± 11.1	0.10
Renal insufficiency	6	1	3	2	0.90
Creatinine (mg dL ${}^{-1}$ )	0.77 ± 0.4	0.82 ± 0.2	0.81 ± 0.4	0.71 ± 0.3	0.32
eGFR <60	6	1	3	2	0.99
Positive blood culture	14	2	7	5	0.13
Condition of the joint					
Healthy	10	0	7	3	0.072 ${}^{d}$
Damaged	31	8	10	13	0.070 ${}^{d}$
AVN	12	2	5	5	0.99
Osteoarthritis	14	4	6	4	0.53
Microbiology					
*S. aureus*	20	2	9	9	0.37
MRSA	5	0	0	5	0.008 ${}^{d}$
Fungus	4	0	1	3	0.40
Polymicrobial	13	1	0	12	0.00010 ${}^{d}$
Final outcome					
Hip preserved	10	0	9	1	0.0023 ${}^{d}$
Hip resected ${}^{a}$	16	0	2	14	0.0023 ${}^{d}$
Hip replaced ${}^{b}$	15	8	6	1	0.000051 ${}^{d}$
Hip replaced later ${}^{c}$	4	0	4	0	0.060 ${}^{d}$
All-time mortality	6	0	2	4	0.27

The abbreviations/acronyms used in the table are as follows: contiguous spreading – CS; hematogenous seeding – HS; direct inoculation – DI; BMI – body mass index; CCI – Charlson comorbidity index; ASA – American Society of Anesthesiology Score; HIV – human immunodeficiency virus; RTx – radiotherapy; SCI – spinal cord injury; CRP – C-reactive protein; WBC – white blood cell count; eGFR – estimated glomerular filtration rate; AVN – avascular necrosis; MRSA – methicillin-resistant *S. aureus*. ${}^{a}$ Hips resected during management of SA *and* not replaced, i.e. Girdlestone procedure and unreplaced antibiotic spacers. ${}^{b}$ Hips that were resected *and* replaced during management of SA. ${}^{c}$ Hips replaced sometime after successful management of SA due to underlying damage to the joint. ${}^{d}$ A statistically significant difference was found.

### 2.4 Statistical analysis

Statistical analysis was performed using R software (version 3.2; R Core Team, 2015). Proportion comparison between categorical variables was performed using a Fisher exact test or chi-square test, depending on the number of observations. Comparison between continuous variables was carried out using a Mann–Whitney U test and Kruskal–Wallis test (in the case of more than two independent variables). Statistical significance was set at p <0.05 .

## 3 Results

### 3.1 Patients

A total of 41 patients with SA of the native hip were identified. The mean patient age was 57.1 years (standard deviation of 17.4; range of 18 to 91), with a male : female ratio of approximately 3:1 (Table 2). The mean length of follow-up was 2.7 years (ranging from 11 d to 14.2 years). The mean Charlson comorbidity index (CCI) and American Society of Anesthesiology Score (ASA) values were 3.2 (standard deviation of 2.4) and 2.6 (standard deviation of 0.7), respectively. No patient had to be excluded due to either an unacceptable risk of surgery or a refusal of therapy. The route of infection was reported to have resulted from DI in 8 patients, HS in 17 patients and CS in 16 patients. Seven patients had multiple joint involvement, with knee (three out of seven) and shoulder (two out of seven) joints being most affected. One patient in the DI group (SA originated from an intra-articular infiltration) developed an active endocarditis secondary to the hip infection. Two patients in the DI group and five patients in the CS group had positive blood cultures. These positive blood cultures were attributed to the hip infections, considering the presence of systemic symptoms and the absence of other primary infections. All results are demonstrated in Table 2.

### 3.2 Three distinct clinical entities of SA of the native hip

#### 3.2.1 Overall fitness

Significant differences in terms of overall fitness were found between the three patient cohorts (Table 2). Most healthy were the patients in the DI group, which had the lowest ASA and CCI scores (2.0 ± 0.8 and 2.0 ± 2.3, respectively) and typically contained patients who developed SA of the hip iatrogenically after an intra-articular injection or as a complication following hip surgery. Less healthy were the patients in the HS group, which had significantly higher ASA and CCI scores (2.6 ± 0.6 and 2.8 ± 2.7, respectively) and comprised a substantial number of patients suffering from SA of more than one joint. Least healthy were the patients in the CS group, which had the highest ASA and CCI scores (2.9 ± 0.3 and 4.2 ± 1.7, respectively) and, in most cases, comprised patients with SA of the hip caused by the progression of decubital wounds on a background of spinal cord injury. Decubital wounds were most often located at the ischial tuberosity (7 out of 16) and/or at the level of the greater trochanter (6 out of 16).

#### 3.2.2 Condition of the hip joint

Although most patients had radiological evidence of some degree of damage to their hip joint at the time of presentation, significant differences were found between the three groups (Table 2). No patients in the DI group had an undamaged hip joint, as all of them had either pre-existing osteoarthritis, avascular necrosis of the femoral head, or a hip or pelvic fracture which was the reason for the intra-articular injection (two out of eight) or the in situ presence of osteosynthesis material (six out of eight) in the first place. One in five patients in the CS group had an undamaged hip joint. For the others, radiological evidence of rapid chondrolysis due to infection was seen at the time of (delayed) presentation, with pre-existing signs of dysplasia, heterotopic ossification and disuse. Two in five patients in the HS group had an undamaged hip joint.

#### 3.2.3 Spectrum of involved micro-organisms

**Figure 1:**
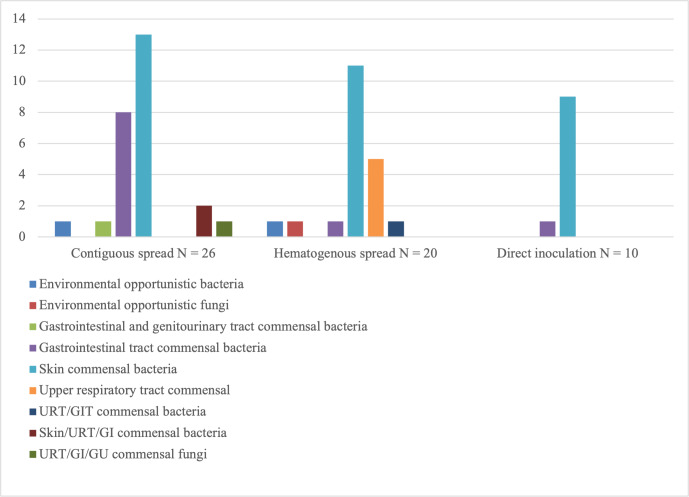
Overview of micro-organisms found per route of infection. Environmental opportunistic bacteria include *Staphylococcus pasteuri* and *Sphingomonas paucimobilis*. Environmental opportunistic fungi include *Aspergillus niger*. Gastrointestinal tract (GIT) and genitourinary tract (GU) commensal bacteria include *Streptococcus agalactiae*. GIT commensal bacteria include *Bacteroides fragilis, Enterococcus faecium, Escherichia coli, Enterobacter cloacae, Citrobacter* species and *Proteus mirabilis*. Skin commensal bacteria include *Staphylococcus aureus, Staphylococcus epidermidis, Staphylococcus capitis, Staphylococcus warneri* and *Corynebacterium* species. Upper respiratory tract (URT) commensal bacteria include *Streptococcus pneumoniae, Streptococcus sanguis, Streptococcus mitis*, and group-C streptococci. URT/GIT commensal bacteria include *Streptococcus anginosus*. Skin/URT/GI commensal bacteria include *Pseudomonas aeruginosa*. URT/GI/GU commensal fungi include *Candida albicans*.

**Figure 2:**
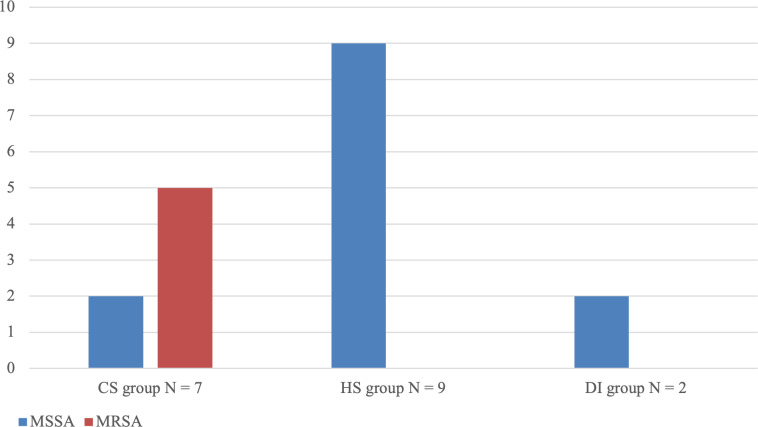
Comparison of methicillin-resistant *Staphylococcus aureus* (MRSA) presence for all three routes of infection. MSSA represents methicillin-sensitive *Staphylococcus aureus*.

Significant differences were found in terms of the type and number of micro-organisms cultured (Table 2). All cases of SA of the native hip in the DI group except one were caused by Gram-positive, aerobic skin commensal bacteria. In one patient, *Bacteroides fragilis* was cultured; in two patients, the infection was poly-bacterial (Fig. 1). Apart from one culture that was positive for *Escherichia coli* and one culture that was positive for *Aspergillus niger*, all SA cases in the HS group were caused by either staphylococci or streptococci. In two patients, more than one micro-organism was identified (Fig. 1). In the CS group, a mix of skin, gastrointestinal and genitourinary tract commensal bacteria was found (Fig. 1). Half of the patients suffered from polymicrobial infections. Methicillin-resistant *Staphylococcus aureus* (MRSA) was only found in this group of patients, with five out of seven cultures positive for *Staphylococcus aureus* showing methicillin-resistance (Fig. 2).

#### 3.2.4 Femoral head preservation

**Figure 3:**
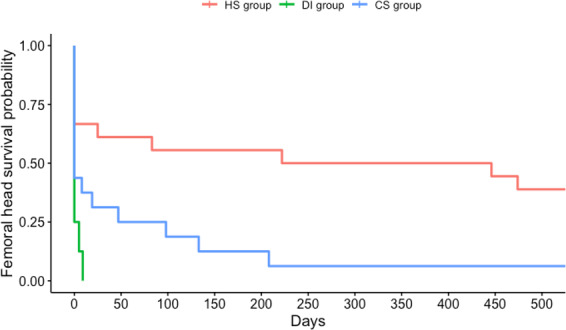
Survival analysis of femoral head preservation per route of infection.

Significant differences were found between the three aforementioned groups (Fig. 3). In all patients in the DI group and all but one patient in the CS group, the femoral head was resected at one point in time. Only patients in the HS group appeared to have had a reasonable chance of retaining their femoral head (9 out of 17). It should be noted that, in the long term, four of these nine patients underwent joint replacement for degenerative disease, and none of them displayed signs of persistent infection at the time of replacement. In these four cases, joint replacement surgery was performed within a median of 16.7 months (ranging from 13.2 to 104.6 months). Noticeably, for eight out of the nine patients in whom the femoral head was retained, a single surgical debridement was sufficient to treat the infection. Most patients (five out of eight) in whom the femoral head was eventually resected, required a second surgical debridement due to inadequate control over the infection. Thus, only patients with SA after hematogenous seeding to an undamaged hip joint had a reasonable chance (53 %) of avoiding joint resection or replacement if control over infection could be achieved without the need for repeated surgical procedures.

#### 3.2.5 Mortality

During the entire follow-up, six patients passed away, equally distributed between the CS and HS groups (mean survival time was 35 months, ranging from 11 d to 84 months). Only one death was directly related to the infection (CS group). This patient passed away due to multi-organ failure only 11 d after the diagnosis of SA of the hip was made.

### 3.3 Duration of symptoms

The duration of symptoms before treatment was uncertain in seven cases (five in the HS group, two in the CS group and none in the DI group). Differences were found between the three groups after removing uncertain cases. Most patients in the DI and CS groups had a delayed presentation and were treated after at least 3 weeks of symptom presentation (6 out of 8 and 12 out of 14, respectively). Most patients in the HS group were treated within 3 weeks of symptom presentation (8 out of 12).

### 3.4 Other prognostic factors

We could not determine any other prognostic factor apart from route of infection ( p<0.05 ) associated with femoral head preservation or infection control within two attempts or less.

### 3.5 Outcome of THA during or after SA of the native hip

THA was performed on 19 patients. Two-stage THA was initiated in 15 cases during active management of SA of the native hip, due to a failure to obtain infection control or because of an extensively damaged hip joint from the start. In four cases, THA was performed a considerable time (range of 13–105 months) after successful treatment of the SA, for secondary degenerative changes. Periprosthetic joint infection (PJI) developed in 2 out of 19 cases (10.5 %). Interestingly, in both cases, a new micro-organism was cultured. Both cases were considered acute PJIs and resolved after a single DAIR (debridement, antibiotics and implant retention) procedure.

## 4 Discussion

SA of the native hip remains one of the few hip disorders for which there are no clear treatment guidelines and for which the outcomes remain relatively uncertain. In this retrospective study, we analysed 41 patients who underwent surgical treatment for SA of the native hip over a period of 16 years. Our findings showed that a single surgical procedure sufficed in less than half of patients and that femoral head resection was deemed necessary in 85 % of cases at some point in time.

The primary objective of our study was to investigate if SA of the native hip should be categorized into three distinct clinical entities based on route of infection. To achieve this, a comprehensive comparison of patient and disease characteristics using the route of infection as the reference point was conducted. Results revealed that there were significant differences among patients with different routes of infection in terms of overall physical fitness, hip joint condition, types of microorganisms involved and the likelihood of retaining the femoral head. In short, patients in the DI group were typically generally healthy individuals who developed SA by skin commensals after an intra-articular injection into an already diseased joint or after hip/pelvis fracture surgery, and all patients ended up with (staged) THA. Patients in the HS group were less healthy patients who developed SA by metastatic spread of staphylococci or streptococci. The involvement of other joints was frequent. Two out of five patients still had healthy hip joints at the time of presentation, and these patients had the highest chance of preserving their femoral head (53 %) if control over infection could be established within two surgical attempts. Patients in the CS group were the least heathy and typically developed poly-bacterial SA (including MRSA) secondary to pressure sores on a background of spinal cord injury. The hips were usually damaged at the time of (delayed) presentation, and resection was almost always deemed necessary.

Therefore, we believe that there are indeed three distinct clinical entities of SA of the native hip joint, and, as we found no other prognostic factors for femoral head resection or the need for repeat surgical debridement, we are also of the opinion that the route of infection should be the basis for clinical decision-making.

The duration of symptoms prior to presentation or surgery could be a major contributor towards treatment outcome. A longer delay could be associated with more damage to the hip joint and a more extensive organization of the infection; however, this will also depend on the type of micro-organism involved. Moreover, as mentioned in Sect. 1, presentation in SA of the native hip joint is often atypical, and it is consequently common that one cannot exactly determine the time of onset. Nevertheless, in our study population, it appears that patients in the HS group were more likely to have an acute presentation resulting in early treatment with better results compared with patients in the DI and CS groups, where treatment was typically delayed ( >3 weeks). However, it is hard to draw significant conclusions from these findings, as the duration of symptoms was uncertain in seven patients (five out of seven patients belonged to the HS group).

We acknowledge that it is difficult to develop a treatment algorithm for these complex patients that can be captured by a single flowchart; however, based on the results of this study, we have adopted the following philosophy for our practice. If we are confronted with a patient with SA of the native hip due to HS, we look at the state of the hip joint and the source of infection. If the hip joint is worth saving, we perform a washout, either open or arthroscopically, based on whether there are extra-articular manifestations of infection on advanced imaging, such as large abscesses, which will also determine the approach to the hip joint in the case of open surgery. We inform the patient that the chance of femoral head preservation will be around 50 %. The number of attempts to save the joint is limited to two, and repeat surgery is only offered after repeat imaging shows that there is no rapid chondrolysis occurring. If the joint is already beyond saving and the source of infection has been dealt with, a one- or two-stage THA is performed, depending on the state of the soft tissues and bone and the presence or absence of difficult-to-treat bacteria. If there is ongoing bacteraemia, however, a washout can be performed to reduce bacterial load to stabilize the patient temporarily.

For patients with SA of the native hip due to DI, the default intervention is a one- or two-stage THA. The exception to this rule is a patient who has had a diagnostic injection into the hip joint, e.g. for a hip-spine dilemma, and in whom diagnosis of SA has been made relatively fast, i.e. within 7 d (Kim et al., 2023). It is important to mention that a significant number of individuals in this population had osteosynthesis material in place. Therefore, there is a possibility of conflation between a native joint SA and fracture-related infection. In such cases, the presence of osteosynthesis material should be taken into consideration when planning the surgical approach.

Patients with SA of the native hip due to CS pose the biggest challenge. If the patient is non-ambulatory a single-stage procedure (definitive Girdlestone) is advocated, which consists of proximal femur resection, filling of the dead space with a muscle flap (typically vastus lateralis) and potentially a hip-bridging external fixator for several weeks (Le Fort et al., 2015; Suda and Heppert, 2010). If the patient is ambulatory, this treatment can be combined with a spacer followed by reimplantation of a THA after the first stage appears successful.

Needless to say, these decisions are made in a multidisciplinary setting, and there are numerous reasons to diverge from one's own treatment principles (Crespo et al., 2020; Whitney et al., 2006).

Our results furthermore show that – seemingly contradictorily – THA appears to be a relatively safe solution, even in these high-risk patients. Our cohort contained 19 patients who underwent a two-stage THA procedure during acute treatment of SA (15 patients) or for its degenerative sequela (4 patients). Two of these patients (10.5 %) re-presented with a PJI, and both settled with a single DAIR procedure. This percentage is comparable to other studies in literature (Bauer et al., 2010; Bettencourt et al., 2022; Chen et al., 2008; Hipfl et al., 2023; Portier et al., 2022; Tan et al., 2021).

It is worth mentioning that our study population showed a significant male predominance, with a male : female ratio of 31:10 . Currently, there are no extensive population-based studies on the epidemiology of native hip SA available. Nonetheless, various smaller studies indicate a marked male predisposition towards this condition (George et al., 2019; Kennedy et al., 2015; Mue et al., 2014; Vassallo et al., 2020). Male : female ratios in these studies ranged from 1.5:1 (George et al., 2019; Mue et al., 2014) up to 5:1 (Vassallo et al., 2020); this variability could be due to demographic differences. Moreover, male predominance was only observed in the HS and CS groups ( 11:6 and 16:0 , respectively). This imbalance could be due to a known male predisposition towards certain underlying illnesses such as endocarditis, decubital ulcers and spinal cord injuries (Baumgarten et al., 2006; DeVivo, 2012; Selton-Suty et al., 2012). In contrast, the DI group showed a balanced male : female ratio of 4:4 , which may be due to the more random distribution of these typically iatrogenic infections. However, even in cases of SA after intra-articular infiltration or arthrocentesis, a higher incidence in males has been reported (Petersen et al., 2019).

This study has its limitations due to its retrospective nature. Firstly, the treatment protocol for femoral head resection was not standardized, leading to potential bias in the surgeon's decision-making. Secondly, despite the fact that the study size is relatively large for studies on SA of the native hip, the sample size is still limited, restricting statistical analysis. Thirdly, some cases may have been missed and medical records may have been incomplete, despite a thorough search of all cultures during the study period. Additionally, some patients were referred from other hospitals, which may have introduced selection bias. Furthermore, it was sometimes difficult to determine the exact duration of symptoms before presentation/surgery. Finally, one could argue that it is not always possible to determine the route of infection with certainty; however, the 41 cases in this study were considered clear-cut.

Further research is needed to verify our findings and objectify better outcomes and a reduced number of surgeries after implementation of our proposed philosophy. Given the small number of cases encountered, even in large hospitals, a multicentre registry would prove useful. Of particular interest to us is the role of advanced imaging, as this might hold important (prognostic) information regarding extra-articular manifestations of the infection and might be able to help select not only the type of treatment but also the surgical approach.

## 5 Conclusion

Patients with SA of the native hip caused by contiguous spreading, hematogenous seeding or direct inoculation differ significantly and should be considered distinct clinical entities. The route of infection is directly related to the possibility of femoral head preservation and should, therefore, be the basis for decision-making. In this study population, only patients with hematogenous infection in a previously healthy hip joint had a possibility of femoral head preservation.

## Data Availability

Raw and anonymized data are available from the corresponding author upon reasonable request.
